# Brazilian Nephrology Census 2019: a guide to assess the quality and scope of renal replacement therapy in Brazil. How are we, and how can we improve?

**DOI:** 10.1590/2175-8239-JBN-2021-E006

**Published:** 2021-06-18

**Authors:** Marcelo Barreto Lopes

**Affiliations:** 1 Arbor Research Collaborative for Health, United States.

The dialysis census of the Brazilian Society of Nephrology in 2019^1^ describes the situation of chronic kidney disease and its impact on the healthcare system in Brazil. Census data helps us improve survival and quality of life for patients undergoing dialysis treatment in Brazil.

Voluntary participation in around 40% of dialysis clinics in the country is an important milestone. The digitized data collection played an important role in the quality of the information obtained. The improvement in data management in the clinics should contribute to further increase the percentage of participation of dialysis clinics in the censuses of the coming years. A high degree of participation in the census - at the moment, greater than participation in the Datasus system - enables clinics to be able to compare treatment quality goals with average metrics at a country level. In addition to its use in clinical practice, the data shown in the publication of the Brazilian dialysis census provide evidence to guide public healthcare policies.

The data show an increase in the prevalence and incidence of patients undergoing dialysis treatment, compared to the previous year. There are regional differences, indicating a higher prevalence where there is greater coverage in the healthcare system. The lower proportion of patients with end-stage kidney disease in states with the lowest per capita income, as are Paraíba and Maranhão, may be attributed to the lower health care coverage for patients with chronic kidney disease in these regions, resulting in less access for patients to early nephrology care treatment and, in particular, vascular access by arteriovenous fistula for maintenance hemodialysis.

The census reports a drop in the mortality of patients undergoing hemodialysis in the country. This reduction has been happening gradually over the years. It would be interesting to document the cause of death in order to understand the factors that contributed to this reduction in mortality, such as, factors associated with better control of infections and better control of cardiovascular diseases in patients.

A worrying finding is the increasing use of long-term catheters and grafts in recent years instead of the use of arteriovenous fistula (AVF). The percentage of arteriovenous fistulas has not been reported. However, with the stability in the proportion of temporary catheter use, it appears that there was no increase in the use of AVF. The data draws attention to the need for a national program similar to the Fistula First program, promoted in the United States^2^. The earlier referral of patients with chronic kidney disease to the nephrologist, together with initiatives that encourage collaboration between nephrologists and vascular surgeons, could contribute to an increase in the percentage of patients with AVF in the beginning of hemodialysis, resulting in better survival of these patients.

From 2005 to 2019, the number of patients on chronic dialysis more than doubled (from 65,129 to 139,691). Estimates indicate that the number of patients on chronic dialysis is expected to continue increasing in the coming years. Data from the United States, for example, indicate that there will be an increase in the prevalence of chronic kidney disease by 2030, regardless of the increase in the prevalence of diabetes and obesity^3^. In Brazil, the prevalence of diabetes and obesity has been increasing with the epidemiological transition. According to data from the Surveillance of Risk Factors and Protection Against Chronic Diseases by Telephone Survey (Vigitel), there was a 34% increase in the prevalence of diabetes between 2006 and 2019^4^. There was also a higher prevalence of systemic arterial hypertension, with a 22% increase during that period. This means that we must prepare for a huge increase in the number of kidney failure patients in the coming years.

The census shows that there is low demand for peritoneal dialysis in Brazil; although most centers have this treatment option, less than 7% of patients end up choosing this mode of therapy. We know that the economic model proposed by our public healthcare system - SUS - is far from what is feasible for most clinics. In the United States (USA), changes in dialysis reimbursement policies have led to an unprecedented growth in the use of peritoneal dialysis (PD) since 2011^5^. Compared to intermittent hemodialysis (HD), PD is more cost-effective and less technically demanding, in addition to being more viable in rural and remote areas^6^, and it is associated with better preservation of residual renal function^7^. This low prevalence of patients on peritoneal dialysis in Brazil can lead to an even greater demotivation in the centers; thus, fewer residents will be trained in the technique - leading to a vicious and damaging cycle for patients with advanced chronic kidney disease.

The SBN dialysis census has been important for the nephrology community, but could improve in the years to come. It is interesting, for example, to obtain data on iron deficiency, regardless of the hemoglobin levels, considering the evidence that this marker is associated with worse outcomes, whether or not there is anemia. Data on patient levels reveal the importance of information on the potassium content used in dialysates in hemodialysis clinics. It will be a great advance to obtain data on the patient's transition in advanced stages of chronic kidney disease to the beginning of hemodialysis, considering the necessary medication adjustments and the preparation of the patient in several aspects for the initiation of renal replacement therapy. The SBN dialysis census is an important tool for improving the quality of treatment of patients with renal failure on renal replacement therapy in Brazil. It is important that the nephrology community uses the census data for the benefit of patients, and supports the census by sending data and communicating the results.
